# COVID-19 inpatients with gastrointestinal onset: sex and care needs’ differences in the district of Ferrara, Italy

**DOI:** 10.1186/s12879-021-06476-y

**Published:** 2021-08-03

**Authors:** Salvatore Greco, Nicolò Fabbri, Alessandro Bella, Beatrice Bonsi, Stefano Parini, Cindy Rocchi, Sara Giaccari, Manuel Gavioli, Angelina Passaro, Carlo V. Feo

**Affiliations:** 1 Department of Translational Medicine, University of Ferrara, Ferrara, Italy - Via Luigi Borsari, 46, 44121 Ferrara, Emilia Romagna Italy; 2 Department of General Surgery, Azienda Unità Sanitaria Locale Di Ferrara, Ospedale del Delta, via Valle Oppio, 2, Lagosanto, 44023 Ferrara, Emilia Romagna Italy; 3 Medical Department, Azienda Unità Sanitaria Locale Di Ferrara, Ospedale del Delta, via Valle Oppio, 2, Lagosanto, 44023 Ferrara, Emilia Romagna Italy; 4 Medical Department, University Hospital of Ferrara Arcispedale Sant’Anna, Via Aldo Moro, 8, 44124 Ferrara, Emilia Romagna Italy; 5 Department of Translational Medicine and Medical Department, University of Ferrara, University Hospital of Ferrara Arcispedale Sant’Anna, Via Aldo Moro, 8, 44124 Ferrara, Emilia Romagna Italy

**Keywords:** SARS-CoV-2, Coronavirus infection, COVID-19, Digestive symptoms, Gastrointestinal onset

## Abstract

**Background:**

COVID-19 is characterized by interstitial pneumonia, but a presentation of the disease with digestive symptoms only may occur.

This work was aimed at evaluating: (1) the prevalence of presentation with digestive symptoms only in our cohort of COVID-19 inpatients; (2) differences between patients with and without gastrointestinal onset; (3) differences among males and females with gastrointestinal presentation; (4) outcomes of the groups of subjects with and without gastrointestinal onset.

**Method:**

We retrospectively divided the patients hospitalized with COVID-19 into two groups: (1) the one with digestive symptoms (DSG) and (2) the other without digestive symptoms (NDSG). We compared the subjects of DSG with those of NDSG and males with females in the DSG group only, in terms of demographics (age, sex), inflammation and organ damage indexes, length of stay, in-hospital and 100-day mortality.

**Results:**

The prevalence of gastrointestinal symptoms at presentation was 12.5%. The DSG group showed a prevalence of females, and these tended to a shorter hospital stay; DSG patients were younger and with a higher load of comorbidities, but no differences concerning inflammation and organ damage indexes, need for intensification of care, in-hospital and 100-day mortality were detected. Among DSG patients, males were younger than females, more comorbid, with higher serum CRP and showed a longer length of hospital stay. Survival functions of DSG patients, in general, are more favourable than those of NDSG if adjusted for sex, age and comorbidities.

**Conclusions:**

(1) The prevalence of gastrointestinal presentation among hospitalized COVID-19 patients was 12.5%; (2) DSG patients were on average younger, more comorbid and with a prevalence of females, with a shorter hospital stay; (3) in the DSG group, males had a higher Charlson Comorbidity Score and needed a longer hospital stay; (4) DSG subjects seem to survive longer than those of the NDSG group.

**Supplementary Information:**

The online version contains supplementary material available at 10.1186/s12879-021-06476-y.

## Background

Coronaviruses are a group of related RNA-viruses that cause diseases in mammals and birds and they are distributed all over the world; in the late 2019, the third member of this family after SARS-CoV-1(Severe Acute Respiratory Syndrome CoronaVirus 1) and MERS-CoV (Middle East Respiratory Syndrome CoronaVirus) appeared for the first time, causing two cases of interstitial pneumonia [1].

SARS-CoV-2 infection can manifest itself in many ways and the set of symptoms (COVID-19, COronaVIrus Disease 2019) ranges from influenza-like symptoms (in 80% of cases) to a severe form of pneumonia (dyspnea or tachypnea with a PaO2/FiO2 ratio < 300 at the blood gas assay and a pulmonary infiltration > 50%). Up to 5% of patients develop rapidly an Acute Respiratory Distress Syndrome (ARDS), with or without acute cardiac damage, related to an underlying systemic hyperinflammation [2, 3] that can hesitate in systemic shock (cytokine storm), multiorgan failure, and death.

Fever is the most frequent sign: it may be absent in up to 50% of patients at the first physical examination, but it seems to be present in more than 90% of cases during hospitalization; cough is present in 67% of cases. Atypical symptoms of systemic involvement can be noticed, such as arthralgia, myalgia, asthenia, headache, upper respiratory tract symptoms, and gastrointestinal symptoms (nausea, vomiting, and diarrhea up to 5% of cases in some experiences, especially during the first periods of pandemic; generalized abdominal pain, anorexia and taste changes were also reported as possible digestive symptoms following SARS-CoV-2 infection) [4, 5].

Further data confirmed that approximately 50% of patients with COVID-19 have detectable viral RNA in the stool [6–8] and the prevalence of gastrointestinal symptoms can be up to 50%. Digestive symptoms were also reported during the epidemics by SARS-CoV-1 and MERS-CoV [9].

As of May 2021, almost 170 million cases of COVID-19 confirmed cases and more than 3.5 million deaths in more than 200 countries and territories were reported (source https://www.worldometers.info/coronavirus/).

The pathophysiology of digestive symptoms associated with COVID-19 remains partially unexplained. It seems that they likely occur because the virus enters the target cells through angiotensin-converting enzyme 2 (ACE2) [10], a receptor found in both the upper and lower gastrointestinal tract where it is expressed at nearly 100-fold higher levels than in respiratory organs [11, 12].

Most studies focused on the respiratory involvement of SARS-CoV-2 infection and the majority of patients with a severe form of COVID-19 faced respiratory symptoms only, without digestive symptomatology [13]. It is likely, however, that a large cohort of undiagnosed patients with a lower severity illness but with digestive symptoms (e.g. diarrhoea, vomiting) may have spread the virus without respiratory involvement [14].

A recent systematic review evaluating a cohort of more than 12,500 patients from 78 studies, found that the pooled prevalence of GI symptoms was 17%; symptoms were evaluated singularly and the weighted prevalence of diarrhea was 12.4%, nausea and/or vomiting 9.0%, loss of appetite 22.3%, and abdominal pain 6.2%. Anyway, the mortality rate among patients with GI symptoms was similar to the overall mortality [15, 16].

A meta-analysis by Hayashi et al. on 4 studies compared COVID-19 patients with and without digestive symptoms; they found abdominal pain to be more frequent in patients with a severe form of disease (OR 2.70), suggesting how this kind of symptom could somehow predict a greater degree of severity of disease [17].

Our study aimed at evaluating differences between patients with and without digestive symptoms in terms of personal, clinical and laboratory parameters; moreover, we tried to understand whether digestive symptoms, in general, could somehow be predictive of different outcomes of COVID-19.

## Materials and methods

This is a two single-center retrospective cohort study. We enrolled 495 adult inpatients (≥ 18 years old) who were consecutively hospitalized with SARS-CoV-2 infection, between March and July 2020, in the “Arcispedale S. Anna” in Cona (Fe) and in the “Ospedale del Delta” in Lagosanto (Fe), the two main hospitals serving the Province of Ferrara. The diagnosis of COVID-19 was determined by at least one SARS-CoV-2 RNA detection at oro- and naso-pharyngeal swab and the presence of symptoms clearly related to the infection itself.

The exclusion criteria were: age (subjects younger than 18 years were excluded), the negativity of swabs to viral detection and/or the positivity of stool cultures to the most common gastrointestinal microorganisms.

The study population was divided into two groups: the “case” group, that was constituted by patients with a gastrointestinal onset and symptoms such as nausea, vomiting and/or diarrhea (Digestive Symptoms Group, DSG) and the “control” group, composed by patients who presented without digestive symptoms at the onset of the disease (Non-Digestive Symptoms Group, NDSG). We did not make any distinction among patients in terms of digestive findings, but we calculated a composite gastrointestinal symptoms’ prevalence.

Patients’ demographic (age, sex), clinical (number of comorbidities) and laboratory data (inflammation and organ damage indexes, samples for viral RNA detection) were extracted from their electronic health records and registered into an electronic database available only to the authors. Data were later anonymized so that no patient could be identified anymore.

We evaluated the load of comorbidities by using the Charlson Comorbidity Index (CCI) which already showed a certain degree of reliability in some studies towards mortality by COVID-19 [18, 19]; the items considered by the CCI are shown in Additional file 1: Table S1.

Inflammation was evaluated by using the white blood cells count (WBC), C-reactive protein (CRP), procalcitonin and ferritin levels; the organ damage was appraised, instead, by using serum creatinine, alanine transferase (ALT), isoamylase, lactic dehydrogenase (LDH) and creatine phosphokinase (CPK); the lymphocytes count was also considered.

The aims of this study was four-fold: (1) to determine the prevalence of gastrointestinal presentation among our cohort of patients hospitalized for COVID-19; (2) to evaluate differences between COVID-19 patients with and without presenting digestive symptoms at presentation, (3) to assess differences among males and females COVID-19 inpatients with gastrointestinal symptoms at presentation, and (4) to evaluate whether the presentation with gastrointestinal onset symptoms is associated with better, indifferent or worse outcomes. We chose as COVID outcomes the following: the need for intensification of care (meant as the need for oro-tracheal intubation or non-invasive ventilation), the in-hospital mortality and the 100-day mortality.

The length of stay was defined as the total number of days spent by the subjects in the hospital setting. With “intensification of care” we defined the need for orotracheal intubation or non-invasive ventilation; the observation period was continued until the 100^th^ day since hospital admission, in order to establish the 100-day mortality rate of our cohort of patients.

We followed STROBE (Strenghtening the Reporting of Observational Studies in Epidemiology) guidelines for reporting observational studies as for the compilation of this manuscript.

The local Ethics Committee (Comitato Etico di Area Vasta Emilia Centro, CE-AVEC), belonging to the “Azienda Ospedaliero-Universitaria” in Bologna, “S.Orsola-Malpighi” Hospital, approved the protocol of this study (code:527/2020/Oss/AUSLFe + AOUFe).

### Statistical analysis

The normal distribution of the continuous variables was analysed using Kolmogorov–Smirnov and Shapiro–Wilk tests. Variables not normally distributed were log transformed before entering parametric statistical analysis. Categorical variables were summarized by using frequencies and percentages, while continuous data were presented as median (interquartile range, IQR).

The Mann–Whitney U test was used for continuous variables, and the χ^2^ test was used for categorical variables. Variables with a *p* value < 0.05 in the univariate analyses were entered into multivariate logistic regression analyses. The Cox regression analyses were performed for evaluating the survival functions of both the groups of patients on statin therapy and not on statin therapy. All *p* values < 0.05 are considered statistically significant.

## Results

Between January and July 2020, 495 adult patients were consecutively admitted at our institutions with SARS-CoV-2 infection. Of these, 62 (12.5%) presented with digestive symptoms only (i.e., nausea, vomiting and or diarrhea), while the remaining patients did not have any gastrointestinal manifestation at the onset of COVID-19.

Table 1 illustrates demographic, clinical, and laboratory data at admission of our patients; these were divided into two groups according to the presentation of disease with (DSG) or without (NDSG) gastrointestinal symptoms. DSG patients were significantly younger than those of NDSG group (70 ± 17 vs. 72 ± 17 years, *p* = 0.043) and with a higher load of comorbidity calculated through their Charlson Comorbidity Index (2 vs. 1 points, *p* = 0.045). The sex distribution was also different between groups, with a greater prevalence of females among DSG patients (62.9% vs. 48.3%, *p* = 0.031), while we found no significant difference between groups in terms of laboratory findings.

**Table 1:** Demographic, clinical, and laboratory findings of patients with COVID-19 on hospital admission, divided for groups (DSG vs. NDSG)

	DSG (N = 62)	NDSG (N = 433)	Overall (N = 495)	*p* value
Sex				0.031
Male, n (%)	23 (37.1)	224 (51.7)	247 (49.9)	
Female, n (%)	39 (62.9)	209 (48.3)	248 (50.1)	
Age, years ± SD	70 ± 17	72 ± 17	72 ± 17	0.042
CCI, points	2 (0–4)	1 (0–3)	1 (0–3)	0.046
White Blood Cells, n/mmc	6040 (5130–9450)	6610 (4935–9585)	6540 (5020–9670)	0.75
Lymphocytes, n/mmc	1085 (785–1485)	1010 (700–1420)	1015 (710–1463)	0.24
CRP, mg/dl	6.6 (2.1–13.2)	5.6 (1.8–11.6)	5.6 (1.9–11.8)	0.31
Procalcitonin, ng/ml	0.22 (0.06–0.43)	0.20 (0.07–0.64)	0.20 (0.07–0.62)	0.98
Creatinine, mg/dl	0.94 (0.77–1.42)	0.97 (0.74–1.28)	0.96 (0.75–1.29)	0.60
Isoamylase, U/L	30 (19–54)	32 (21–51)	32 (21–52)	0.92
ALT, U/L	19 (14–34)	23 (14–37)	22 (14–36)	0.51
LDH, mg/dl	282 (202–410)	268 (199–363)	269 (200–368)	0.31
CPK, U/L	86 (48–181)	95 (46–179)	89 (46–178)	0.73

Data are reported as median (IQR) unless otherwise specified

*DGS* Digestive Symptoms group, *NDGS* No Digestive Symptoms Group, *SD* Standard Deviation, *CCI* Charlson Comorbidity Index, *CRP* C Reactive Protein, *ALT* Alanine Transferase, *LDH* Lactic Dehydrogenase, *CPK* Creatine Phosphokinase

Comorbidities were evaluated separately (Table 2) and the only significant differences between DSG and NDSG groups concerned the percentage of patients with heart failure (1.6% vs. 12.7%, *p* = 0.009) and chronic obstructive pulmonary disease COPD (0% vs. 10.4%, *p* = 0.008): anyway, such data cannot be considered consistent because of the small size of the sample involved.

**Table 2:** Comorbidities on admission, divided for groups (DSG vs. NDSG)

Comorbidity	DSG (N = 62)	NDSG (N = 433)	*p* value
Hypertension	41 (66.1)	265 (61.2)	0.52
Diabetes	19 (30.6)	89 (20.6)	0.07
Ischemic Heart Disease	5 (8.1)	58 (13.4)	0.23
Heart Failure	1 (1.6)	55 (12.7)	0.009
Chronic Kidney Disease (III-IV-V stage)	8 (12.9)	55 (12.7)	1.00
Stroke or TIA	9 (14.5)	52 (12.0)	0.84
PCOA	5 (8.1)	23 (5.3)	0.38
COPD	0 (0)	45 (10.4)	0.008
Hepatic Steatosis	3 (4.8)	9 (2.1)	0.19
End-stage Liver Disease	2 (3.2)	6 (1.4)	0.29
Localized or Hematological Cancer	15 (24.2)	70 (16.2)	0.13
Metastatic Cancer	6 (9.7)	21 (4.8)	0.12

All data are expressed with number of cases (%)

*DGS* Digestive Symptoms group, *NDGS* No Digestive Symptoms Group, *TIA* Transitory Ischemic Attack, *PCOA* Peripheral Chronic Obstructive Arteriopathy, *COPD* Chronic Obstructive Pulmonary Disease

None of the subjects, among our patients, suffered from inflammatory bowel diseases (IBD), neither in the DSG group, nor in the NDSG one; among DSG subjects, 3 had hepatic steatosis in their clinical history (4.8%) and 2 an end-stage liver disease (3.2%). 17 patients of the DSG group (27.4%) took protonic pump inhibitors (PPI) before hospital admission; moreover, all subjects in the two groups were of Caucasian origin.

DSG patients showed a tendency towards a shorter duration of hospital stay (18 ± 13 days vs. 20 ± 15 days, *p* = 0.038), while we found no significant differences between DSG and NDSG patients concerning the three outcomes of disease chosen. ((1) The need for intensification of care; (2) The in-hospital death; (3) The 100-day death), as shown in Table 3.

**Table 3:** Clinical outcomes differences between groups (DSG vs. NDSG)

	DSG (N = 62)	NDSG (N = 433)	Overall (N = 495)	*p* value
Length of Stay, days ± SD	18 ± 13	20 ± 15	19 ± 15	0.038
Intensification of Care, n (%)	14 (22.6)	108 (24.9)	122 (24.6)	0.69
In-hospital death, n (%)	13 (21.0)	108 (24.9)	121 (24.4)	0.50
100-day Death, n (%)	18 (29.0)	132 (30.5)	150 (30.3)	0.82

Data are reported as number of subjects (%) unless otherwise specified

*DGS* Digestive Symptoms group, *NDGS* No Digestive Symptoms Group, *SD* Standard Deviation

Basing on the different distribution of males and females between groups, we decided to perform new analyses concerning demographic, clinical and laboratory data in the DSG group. For each parameter, we searched for significant differences between males and females (Table 4).

**Table 4:** Demographic, clinical, and laboratory findings in males and females in the Digestive Symptoms Group (DSG)

	DSG (N = 62)		*p* value
	Males (N = 23)	Females (N = 39)	
Age, years ± SD	66 ± 18	72 ± 17	0.022
CCI, points	2 (1–5)	1 (0–1)	0.034
White Blood Cells, n/mmc	6040 (5130–9650)	6140 (5640–9140)	0.60
Lymphocytes, n/mmc	985 (380–1000)	1155 (1180–1890)	0.38
CRP, mg/dl	11.1 (9.4–21.3)	4.9 (2.0–8.0)	0.043
Procalcitonin, ng/ml	0.28 (0.10–5.83)	0.18 (0.02–0.43)	0.14
Creatinine, mg/dl	1.09 (0.79–1.46)	0.86 (0.75–1.24)	0.22
Isoamylase, U/L	32 (19–46)	30 (23–33)	0.99
ALT, U/L	22 (15–42)	18 (13–23)	0.25
LDH, mg/dl	369 (209–425)	246 (183–296)	0.07
CPK, U/L	88 (63–187)	63 (86–134)	0.34

Data are reported as median (IQR) unless otherwise specified

*DGS* Digestive Symptoms group, *NDGS* No Digestive Symptoms Group, *SD* Standard Deviation; CRP, C Reactive Protein, *ALT* Alanine Transferase, *LDH* Lactic Dehydrogenase, *CPK* Creatine Phosphokinase

Females, in particular, tended to a shorter length of stay (17 ± 13 vs. 20 ± 14 days, *p* = 0.011), while there were not substantial differences between sexes in terms of need for intensification of care, in-hospital death and death after 100 days. These findings are reported in Table 5.

**Table 5:** Clinical outcomes measure of patients in the Digestive Symptoms Group (DSG): Males vs. Females

	Males (N = 23)	Females (N = 39)	Overall (N = 62)	*p* value
Length of Stay, days ± SD	20 ± 14	17 ± 13	18 ± 13	0.011
Intensification of Care, n (%)	7 (30.4)	7 (17.9)	14 (22.6)	0.26
In-hospital death, n (%)	5 (21.7)	8 (20.5)	13 (21.0)	0.91
100-day Death, n (%)	6 (26.1)	12 (30.8)	18 (29.0)	0.70

Data are reported as number of subjects (%) unless otherwise specified

*DGS* Digestive Symptoms group, *NDGS* No Digestive Symptoms Group, *SD* Standard Deviation

Logistic regression analyses were also performed choosing those variables that confirmed to be statistically different between groups; we tried to understand which of the factors considered in our work could somehow independently determine a better or a worse prognosis in our cohort of patients. Anyway, none of the variables tested resulted to be independently associated with COVID-19 outcomes (data not shown).

**Fig. 1:**
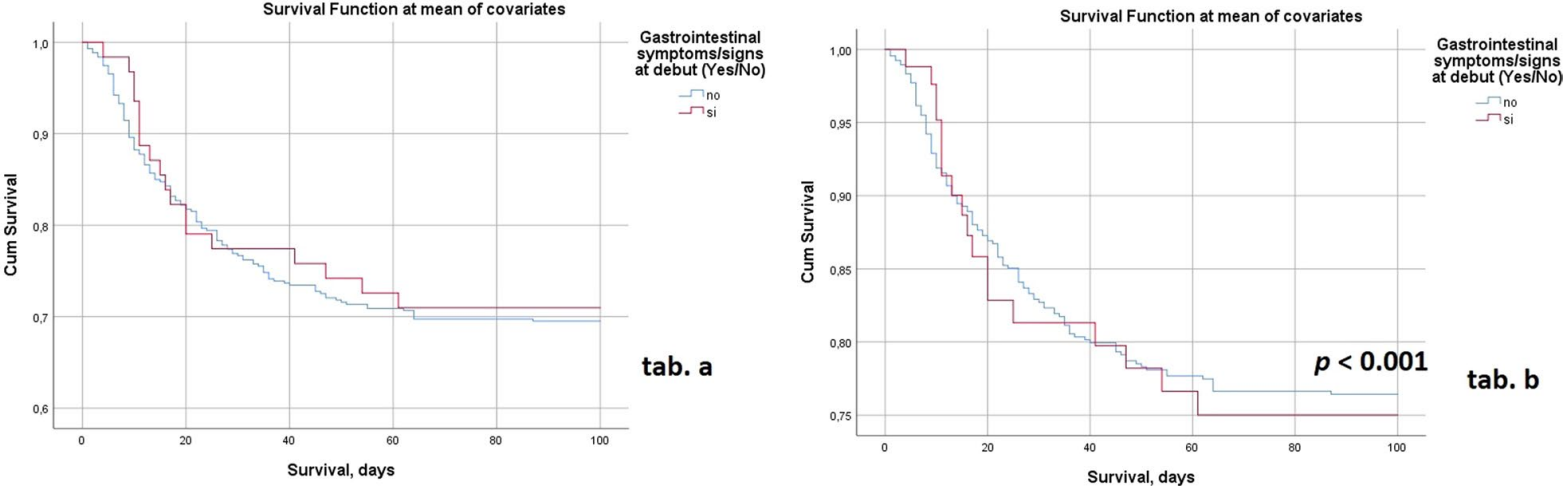
Cox regression analyses, 100-day survival functions in both groups of patients (Gastrointestinal symptoms/signs at debut = red curve vs. No gastrointestinal symptoms/signs at debut = blue curve). Table a shows the survival curves of the two groups, without any adjustment. In table b, the same Cox regression analysis with adjustment for sex, age and CCI (Charlson Comorbidity Index): the red curve intercepts the blue curve in two descending tracts, but curves take different directions after a longer observation time, resulting in two different survival functions

To understand the role of the variables sex, age and CCI, supposing them to be the main selection bias of our findings, we decided to perform two separate Cox regression analyses, that determined the survival curves of the population with and without gastrointestinal onset (DSG and NDSG, respectively). The Cox regression analysis in Fig. 1, tab. a, shows the survival curves of both populations after 100 days of observation without any adjustment. In tab. b of Fig. 1 it is shown the Cox regression analysis for the same populations of patients but with the adjustment for sex, age and comorbidities (weighted through the CCI): the population of patients with digestive symptoms only at the debut of COVID-19 seems to survive longer if compared to that of patients without gastrointestinal onset (*p* < 0.001).

## Discussion

In this study, we enrolled 495 patients, consecutively hospitalized to our two hospitals of Ferrara’s territory dedicated to COVID-19 inpatients. Among the patients hospitalized for SARS-CoV-2 infection, the prevalence of gastrointestinal symptoms at presentation was 12.5%. COVID-19 patients presenting with digestive symptoms only, as opposed to those without, showed a prevalence of females, and these ones tended to a shorter hospital stay.

One of the first case series published in China on 40 COVID-19 inpatients reported that the main symptom was dyspnoea (55%) while diarrhoea was described in 3% of cases only; the median age of subjects was 49 years [1]. Other Chinese studies reported different percentage of gastrointestinal symptoms [20, 21]: Zhang et al. reported fever (91%), cough (75%), fatigue (75%) and gastrointestinal symptoms (39%) as the most common clinical manifestations in 140 hospitalized COVID -19 patients while Wang et al. found a gastrointestinal onset rate of 10.1% in a group of 138 hospitalized patients with a median age of 56 years. Cheung et al., in their meta-analysis of 60 studies involving 4,243 COVID-19 patients from six countries, found digestive symptoms in 17.6% of the patients with specific rates of anorexia (26.8%), diarrhoea (12.5%), nausea/vomiting (10.2%) and abdominal pain/discomfort (9.2%). In this meta-analysis, authors suggested that gastrointestinal symptoms are more common in the severe form of the disease (17.1%) [22].

In a meta-analysis on 118 international subjects by Sultan et al., evaluating also subjects outside of China, gastrointestinal symptoms were reported in 10% of cases, while ALT increases were found in 15–20% of the subjects involved in the analysis. Diarrhea was present in 6.2% of all inpatients from China (8.9% in the overall analyses, regardless of hospitalization), while as for non-Chinese patients these percentage was much higher (18.9% among the inpatients, 20.1% in the overall population); analyses were performed also considering diarrhea as presenting symptoms of the disease with a percentage of 9.3% in the overall population (8.0% among Chinese subjects and 20.0% in the non-Chinese cohort). Nausea and/or vomiting were reported in 7.6% of the overall population, regardless of hospitalization (4.9% of Chinese subjects, 15.9% among non-Chinese subjects); abdominal pain was, instead, present in 4.0% of patients (3.0% among Chinese subjects, 6.4% among non-Chinese subjects) [23]. Such data could suggest a sort of regional difference in the predisposition to digestive symptoms, according to which the Chinese population would manifest COVID-19 in a lower percentage of cases with gastrointestinal onset. Studies concerning this aspect are still missing and it is still impossible to make any kind of conclusion about it. Some studies were also performed in the pediatric population and a very recent meta-analysis by Bolia et al. [24] evaluated the prevalence rates of diarrhea, nausea/vomiting and abdominal pain among young subjects: such symptoms were reported to be present in nearly 20% of patients and were associated with the severe clinical form of COVID-19.

In order to compare the outcomes of patients in both DSG and NDSG groups, we evaluated the duration of length of hospital stay, the need for intensification of care, the in-hospital death and the global 100-day death. COVID-19 patients presenting with digestive symptoms tended to have a shorter hospital stay, but showed no difference concerning the major outcomes of the disease, differently from the studies cited above. In addition, in the DSG there was a prevalence of females (62.9% vs. 48.3%) and, for this reason, we decided to extend our analyses to search for differences between males and females in terms of age, comorbidities (through the CCI score), inflammation and organ damage indexes, finding males to be younger than females (median age 66 ± 18 vs. 72 ± 17 years), on average more comorbid and with higher C-reactive protein levels. No differences among sexes were found in terms of COVID-19 outcomes, even if female inpatients tended to a shorter length of hospital stay.

The debut of disease with gastrointestinal symptoms was initially associated with a better prognosis of COVID-19, and usually intended as a mild or moderate illness [6, 8]. The opposite was observed in the population with a respiratory presentation of the disease, which could go towards mild, moderate or severe diseases depending on the case [13].

Further analyses evaluating a greater number of subjects showed completely different associations, as already stated above, with digestive symptoms more often associated to worse outcomes, in general. Many hypotheses were made about this unfavourable association by the gastrointestinal onset; the most accredited hypothesis states that the debut of the disease at a digestive level would somehow delay the hospital admission, indirectly allowing a greater viral intracellular replication, leading to a late treatment and, thereby, a worse prognosis. However, whether intestinal lesions of COVID-19 are the result of a secondary response after systemic inflammation, are the result of a primary intestinal infection, or are the combined results of both mechanisms remains uncertain.

Another interesting point of view concerns the sex differences found in this study. Previous works showed that females, due to higher innate cellular and humoral immune responses, react differently than males to both viral infections and vaccines [25]. Other authors supported the idea of different sex-specific responses to viral infections during acute virological control and in immunopathologic manifestations [26].

It is possible that female sex gives some kind of immune protection against SARS-CoV-2, as it happens for other viruses, through complex interactions between sex hormones, sex chromosomes, and immune response genes. This could lead to a different presentation of disease in males that could partially explain the difference in prevalence among females and males in our population with gastrointestinal presentation.

The survival functions of DSG and NDSG groups showed that the patients with gastrointestinal onset have a more favourable prognosis of the disease after 100 days. This could have been partially explained by the sort of “protection” that the female sex gives towards SARS-CoV-2 infection. For this reason, we decided to adjust the COX regression analysis for sex, as well as for age and comorbidities, with significant results, particularly in contrast with some of the findings previously cited.

It is still unclear why the gastrointestinal colonization by SARS-CoV-2 effectively affects the prognosis of patients, nor how this could happen. Thus, despite studies about this specific aspect of disease are day by day increasing, it is necessary to further investigate the clinical aspects of COVID-19 patients presenting with gastrointestinal symptoms.

This study has several limitations, and our findings must be read with caution. The main limitations of this work are attributable, in the first instance, to its retrospective nature and the inclusion of a limited number of COVID-19 patients presenting with digestive symptoms. Moreover, it was not possible to evaluate singularly all the possible etiologic factors underlying the gastrointestinal onset of the disease: the small size of sample, in fact, did not allow to perform specific analyses for each of them.

## Conclusions

The prevalence of digestive symptoms at the debut of COVID-19 in our cohort of inpatients was 12.5%. In the group of patients with gastrointestinal onset (DSG) there was a greater prevalence of females and subjects were significantly younger but with a higher load of comorbidities (this was expressed through the evaluation of the Charlson Comorbidity Score, CCI). Exploring the differences between sexes among DSG patients, we found that males were younger than females, with a greater CCI score, and with higher serum C-reactive protein; moreover, females tended to a shorter length of stay than males, while there was no difference in terms of prevalence concerning the major outcomes of the disease.

COX regression analyses showed more favourable survival functions after 100 days by the DSG patients, if adjusting the test for age, sex and comorbidities.

## Supplementary Information

**Additional file 1: Table S1.** The components of the Charlson Comorbidity Index.

## Data Availability

Data are protected by a research consortium. The Corresponding Author will share the data upon formal proposal of the interested researchers.
