# Supplementary material for: COVID-19 inpatients with gastrointestinal onset: sex and care needs’ differences in the district of Ferrara, Italy
Source: BMC Infect Dis. 2021 Aug 3;21:739. doi: 10.1186/s12879-021-06476-y (PMC8329637; doi:10.1186/s12879-021-06476-y)
Supplement: Supplementary file 1 — Additional file 1: Table S1. The components of the Charlson Comorbidity Index. [file 12879_2021_6476_MOESM1_ESM.docx]

**Table S1. The components of the Charlson Comorbidity Index**

| **Weight** | **Conditions** |
| --- | --- |
| 1 point | Myocardial infarction, Congestive heart failure, Peripheral vascular disease, Cerebrovascular disease, Dementia, Chronic obstructive disease, Connective tissue disease, Ulcer disease, Mild liver disease, Diabetes mellitus |
| 2 points | Hemiplegia, Moderate/severe renal disease, Diabetes with end-stage organ damage Any tumor, Leukemia, Lymphoma |
| 3 points | Moderate/severe liver disease |
| 6 points | Metastatic solid tumor, AIDS |
